# Selective Impedimetric Chemosensing of Carcinogenic Heterocyclic Aromatic Amine in Pork by dsDNA-Mimicking Molecularly Imprinted Polymer Film-Coated Electrodes

**DOI:** 10.1021/acs.jafc.1c05084

**Published:** 2021-11-29

**Authors:** Viknasvarri Ayerdurai, Alvaro Garcia-Cruz, Joanna Piechowska, Maciej Cieplak, Paweł Borowicz, Krzysztof R. Noworyta, Grzegorz Spolnik, Witold Danikiewicz, Wojciech Lisowski, Agnieszka Pietrzyk-Le, Francis D’Souza, Wlodzinierz Kutner, Piyush Sindhu Sharma

**Affiliations:** † Institute of Physical Chemistry, Polish Academy of Sciences, Kasprzaka 44/52, 01-224 Warsaw, Poland; ‡ Institute of Organic Chemistry, Polish Academy of Sciences, Kasprzaka 44/52, 01-224 Warsaw, Poland; § Department of Chemistry, University of North Texas, 1155 Union Circle No. 305070, Denton, Texas 76203-5017, United States; ∥ Faculty of Mathematics and Natural Sciences, School of Sciences, Cardinal Stefan Wyszynski University in Warsaw, Wóycickiego 1/3, 01-938 Warsaw, Poland

**Keywords:** heterocyclic aromatic amine, quinoxaline, impedimetric chemosensor, molecularly imprinted polymer, MIP, nucleobase bithiophene derivative, intercalation, allosteric recognition, molecular recognition, bio-mimicking material

## Abstract

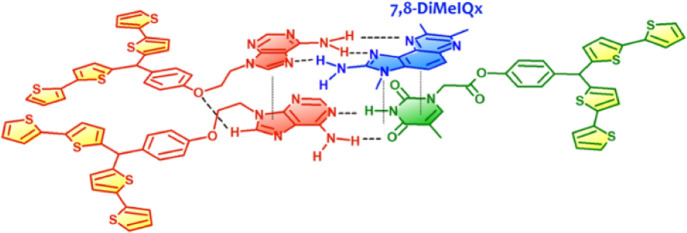

Inspired by the easy intercalation of quinoxaline heterocyclic aromatic amines (HAAs) in double-stranded DNA (dsDNA), we synthesized a nucleobase-functionalized molecularly imprinted polymer (MIP) as the recognition unit of an impedimetric chemosensor for the selective determination of a 2-amino-3,7,8-trimethyl-3*H*-imidazo[4,5-*f*]quinoxaline (**7,8-DiMeIQx**) HAA. HAAs are generated in meat and fish processed at high temperatures. They are considered to be potent hazardous carcinogens. The MIP film was prepared by potentiodynamic electropolymerization of a pre-polymerization complex of two adenine- and one thymine-substituted bis(2,2′-bithien-5-yl)methane functional monomer molecules with one **7,8-DiMeIQx** template molecule, in the presence of the 2,4,5,2′,4′,5′-hexa(thiophene-2-yl)-3,3′-bithiophene cross-linking monomer, in solution. The as-formed MIP chemosensor allowed for the selective impedimetric determination of **7,8-DiMeIQx** in the 47 to 400 μM linear dynamic concentration range with a limit of detection of 15.5 μM. The chemosensor was successfully applied for **7,8-DiMeIQx** determination in the pork meat extract as a proof of concept.

## 1 Introduction

**Scheme 1:**
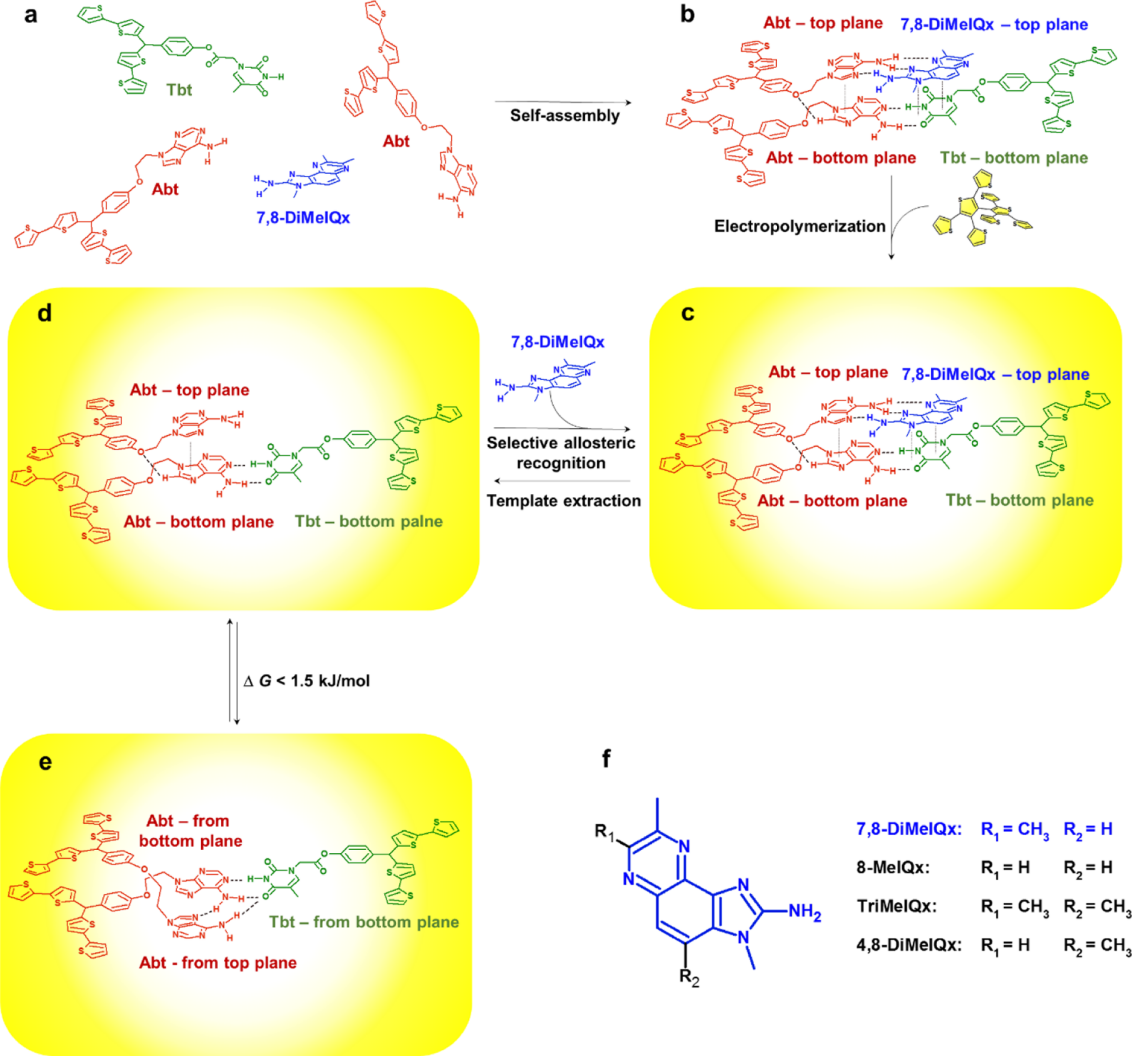
Structural Formulas of (a) **Abt and Tbt** Functional Monomers and the **7,8-DiMeIQx** Template, (b) the **Abt–Tbt/Abt**-(**7,8-DiMeIQx**) Pre-polymerization Complex, and (c–e) the Imprinted Molecular Cavity in the MIP Film (c) with the Template Molecule before Extraction and (d, e) after Template Extraction in the (d) Ready for Analyte Recognition and (e) Inactive Conformations; (f) Structural Formulas of 2-Amino-3,7,8-trimethyl-3*H*-imidazo[4,5-*f*]quinoxaline (**7,8-DiMeIQx**) and Its Close Analogues 2-Amino-3,8-dimethylimidazo[4,5-*f*]quinoxaline (**8-MeIQx**), 2-Amino-3,4,7,8-tetramethyl-3*H*-imidazo[4,5-*f*]quinoxaline (**TriMeIQx**), and 2-Amino-3,4,8-trimethyl-3*H*-imidazo[4,5-*f*]quinoxaline (**4,8-DiMeIQx**)

Quinoxaline heterocyclic aromatic amines, HAAs, are mutagens and potent carcinogens generated in meat and fish processed at high temperatures.^1−3^ Several classes of HAAs have been identified. Heat-processed food of animal origin causes creatinine condensation with hexoses, pyrazine, and pyridine derivatives to form aminoimidazoarenes. Because of their widespread occurrence in the diet and the environment, HAAs cause several common stomach, colorectal, pancreatic, and breast cancer diseases. Notably, HAAs are not cancerogenic by themselves. However, they undergo enzymatic transformations inside the organism.^1,4^ The nitrenium and diazonium ions, produced as HAA metabolites, readily intercalate double stranded DNA (dsDNA) and covalently bind with nucleobases.^5−7^ Moreover, the resulting adduct structure is stabilized by a hydrogen bond with the oxygen atom of the 5′-phosphodiester linkage. The HAA intercalation results in nucleobase displacement in the dsDNA structure. Herein, we focus on the first HAA class represented by 2-amino-3,7,8-trimethyl-3*H*-imidazo[4,5-*f*]quinoxaline, viz., **7,8-DiMeIQx** (Scheme 1).

HAAs are present in the food of animal origin at a very low concentration (∼ng per g of a food sample)^4,8,9^ and, therefore, are difficult to determine in food matrices. Currently, this challenging determination is performed using rather complex procedures involving high-performance liquid chromatography preceded by pre-concentration/isolation using different techniques.^3,4,8−16^ Moreover, fluorescence spectroscopy was used for **4,8-DiMeIQx** (Scheme 1) determination with carbon dots.^17,18^ However, the results obtained were pH-dependent and, moreover, in both reports, the detectability of the analytical system was insufficiently low. That is, the reported limit of detection (LOD)^17^ and limit of quantification^18^ were very high, namely, equal to 1.3 and 0.36 mg L^–1^, respectively. These values were far above the average concentration of HAAs in food.

Nowadays, molecularly imprinted polymers (MIPs) are broadly used to devise artificial recognition systems by mimicking the recognition features of the corresponding biological receptors.^19^ Furthermore, chemosensors using MIPs have become remarkably widespread because of their selectivity, appreciable chemical and mechanical stability, as well as relatively inexpensive and straightforward preparation.^20^ Therefore, numerous applications of MIP chemosensors in food product analysis were reported.^21,22^ MIPs have successfully mimicked the natural receptors and, therefore, were applied to sensing devices as recognition units for selectively determining dsDNA.^23,24^ Moreover, MIPs capable of DNA mimicking were proposed. For instance, ssDNA of a six-nucleotide sequence was imprinted in polythiophene-containing monomers modified with nucleobases.^25^ The resulting MIP selectively recognized the target ssDNA by Watson–Crick nucleobase pairing. MIPs bearing adenine functional monomers were employed in another approach to determine 5-fluorouracil, an antitumor drug,^26^ and thalidomide,^27^ an immunomodulatory drug. Likewise, MIPs with cytosine-substituted bis-bithiophenes were used for the selective recognition of 6-neopterin^28^ and 6-thioguanine.^29^ However, all these compounds were recognized by MIP films using only hydrogen bond patterns. There is no example of a DNA-mimicking MIP film applied for the recognition of intercalating molecules, to our best knowledge.

To this end, we propose a novel dsDNA-mimicking MIP film recognizing a **7,8-DiMeIQx** cancerogenic intercalator with similar interactions as those involved in real dsDNA intercalation. The chemosensor recognizing unit devised in the present study engaged an MIP film prepared using bioinspired nucleobase artificial functional monomers (Scheme 1), capable of both Watson–Crick^30^ nucleobase pairing and stable Hoogsteen-type hydrogen bonding, like that in drug interactions with dsDNA.^5^ Herein, molecular cavities mimicking allosteric molecular recognition^31^ in biological receptors were uniquely designed to reach high selectivity for quinoxaline HAA determination. That is, our dedicated supramolecular polymeric receptors, synthesized by molecular imprinting, were present in two different interconvertible conformations remaining in thermal equilibrium. The target analyte was capable of binding to the receptor only in one of these conformations, thus shifting the equilibrium toward the desired product (Scheme 1). Therefore, the MIP revealed a very high selectivity. Furthermore, to highlight possible future applications of the devised MIP–chemosensor in food safety control, we applied it successfully for **7,8-DiMeIQx** determination in pork meat samples.

## 2 Experimental Section

### 2.1 Computational Calculations

Structures of the pre-polymerization complex of **7,8-DiMeIQx** with 4-bis(2,2′-bithien-5-yl)methylphenyl 2-adenine ethyl ether (**Abt**) and 4-bis(2,2′-bithien-5-yl)methylphenyl thymine-1-acetate (**Tbt**) functional monomers were optimized, and the changes in thermodynamic functions due to pre-polymerization complex formation were calculated using density functional theory (DFT) at the M06-2X/6-31G* level with dispersion correction (D3) using Gaussian 09 software^32^ on a high-speed PC.

### 2.2 Chemicals

Acetonitrile, isopropanol, toluene, used in both electrochemical experiments and chemical syntheses, and triethylamine (Et_3_N) were purchased from Sigma-Aldrich. HAAs, namely, 2-amino-3,8-dimethylimidazo[4,5-*f*]quinoxaline (**8-MeIQx**) and 2-amino-3,7,8-trimethyl-3*H*-imidazo[4,5-*f*]quinoxaline (**7,8-DiMeIQx**) were procured from Toronto Research Chemicals (TRC). 4-Bis(2,2′-bithien-5-yl)methylphenyl 2-adenine ethyl ether (**Abt**)^25,26^ and 4-bis(2,2′-bithien-5-yl)methylphenyl thymine-1-acetate (**Tbt**)^25^ functional monomers as well as the 2,4,5,2′,4′,5′-hexa(thiophene-2-yl)-3,3′-bithiophene (**Crl**)^25,33^ cross-linking monomer were prepared according to procedures described previously. For solution preparation, deionized (18.2 MΩ cm) Milli-Q water (EMD Millipore, Billerica MA, USA) was used.

### 2.3 Instrumentation

Electrochemical experiments were performed with an SP-300 electrochemistry system of Bio-Logic Science Instruments. EC-Lab version 10.37 software of the same manufacturer was used to control this system. A ∼0.5 mL three-electrode one-compartment V-shaped glass vessel served as the electrochemical minicell. A 1 mm diameter Pt disk sealed in a soft glass tubing, a silver wire, and a coiled Pt wire were used as the working, quasi-reference, and auxiliary electrodes, respectively, in the potentiodynamic, cyclic voltammetry, differential pulse voltammetry (DPV), and electrochemical impedance spectroscopy (EIS) experiments. Before each electropolymerization, the Pt working electrodes were cleaned for 30 s in a “piranha” solution, H_2_O_2_:H_2_SO_4_, 1:3 (v/v). (Warning: the “piranha” solution is dangerous if it comes in contact with the skin or eyes because it reacts violently with most organic compounds).

Polymer films were characterized by atomic force microscopy (AFM) imaging, high-resolution X-ray photoelectron spectroscopy (XPS), and Fourier-transform infrared (FTIR) spectroscopy. For that purpose, the films were deposited on ∼0.5 cm^2^ area Au-layered glass slides. These slides were cleaned with acetone and then dried in an Ar stream before use.

For imaging, a MultiMode 8 AFM microscope with a Nanoscope V controller (Bruker) was used in the tapping mode, with high-sensitivity and quality-etched silicon RTESPA-300 (40 N/m) probes (Bruker), controlled by Nanoscope v. 8.15 software (Bruker). MIP and non-imprinted (NIP) film samples for AFM imaging were gently scratched in selected places with polyethersulfone (PES) tweezers to determine the polymer film thickness. The films were scratched just after polymerization, that is, until the films remained wet and soft. That way, some part of the film was removed, and a gold electrode substrate surface was exposed. Then, the height of the step formed was determined by imaging in 5 to 10 spots, sufficiently far from a partially detached film edge. Then, the results of the measurements were averaged.

The high-resolution XPS experiments were performed with a PHl5000 VersaProbe-Scanning ESCA Microprobe instrument at a base pressure of below 5 × 10^–9^ mbar. Monochromatic Al Kα radiation was used, and the X-ray beam, focused to 100 μm diameter, was scanned over a (250 × 250) μm^2^ sample surface at an operating power of 25 W (15 kV).

The FTIR spectra of the deposited MIP and NIP films were recorded in the reflection mode with a Vertex 80v spectrophotometer of Bruker using a deuterated triglycine sulfate detector controlled by OPUS v. 6.5 software.

### 2.4 Procedures

#### 2.4.1 Synthesis and Deposition of MIP and NIP Films

An MIP was synthesized and simultaneously deposited as a thin film on the electrode surface by potentiodynamic electropolymerization from the acetonitrile solution of 20 μM **7,8-DiMeIQx**, 40 μM **Abt**, 20 μM **Tbt**, 40 μM **Crl**, and 20 mM (TBA)ClO_4_ —a supporting electrolyte. The MIP film was deposited by performing two potential cycles from 0 to 1.25 V and back to 0 V versus an Ag quasi-reference electrode. The scan rate was 50 mV s^–1^. The three-electrode system described in Section 2.3 above was used for studying that process. After electropolymerization, the MIP film-coated electrode was triply rinsed with acetonitrile. Finally, the **7,8-DiMeIQx** template was removed from the MIP by 20 min extraction with 10 mM Et_3_N in acetonitrile.

A control NIP film was prepared following the same electropolymerization and “extraction” procedures but in the absence of **7,8-DiMeIQx**.

The MIP and NIP films were deposited on Au-layered glass slides for XPS, FTIR, and AFM characterization, using the same procedures as those described above.

#### 2.4.2 Electrochemical Measurements

In the EIS studies, the potential was kept constant at 0.21 V versus the Ag quasi-reference electrode. At this potential, the Faradaic process of the 100 mM K_3_[Fe(CN)_6_] and 100 mM K_4_[Fe(CN)_6_] redox probe in phosphate-buffered saline (PBS) (pH = 7.4) was the most pronounced. The EIS experimental data were fitted with EC-lab V10.44 software from Bio-Logic. The modified Randles–Ershler equivalent electric circuit selected for mimicking the electrode-solution interface comprised a solution resistance (*R*_s_) in series with the parallelized constant-phase element in series connected with the impedance of the Faradaic reaction charge transfer resistance (*R*_ct_) and Warburg impedance (*W*) describing diffusion processes.^34^ The EIS spectra were recorded in the 100 mHz to 200 kHz frequency range at the 10 mV ac amplitude. In this range, all studied electrode processes proceeded.

For DPV determinations, the potential scan range was 0 to 0.50 V versus the Ag quasi-reference electrode, the potential step was 5 mV, the pulse amplitude was 50 mV, and the pulse duration was 50 ms.

The capacity measurements were performed both under the steady-state and flow-injection analysis (FIA) conditions. The electrochemical cell used for the former was the same as that used for the DPV and EIS experiments. The FIA measurements were performed using a large-volume (∼35 mL) radial-(thin-layer) flow electrochemical cell^35^ filled with the carrier solution. The 1 mm diameter Pt disk working electrode was axially mounted, opposite to the inlet capillary, at a (capillary outlet)-to-electrode distance of 500 μm. A Pt wire loop and an Ag wire were used as the auxiliary and quasi-reference electrodes, respectively. The analyte samples were dissolved in the solution of the same composition as that of the carrier liquid, that is, 10 mM KF in deionized water. The applied potential was *E* = 0.21 V versus the Ag quasi-reference electrode with a 10 mV amplitude of *f* = 500 Hz frequency. At this potential, no Faradaic process occurred.

Calibration plots for **7,8-DiMeIQx** in the same concentration range using MIP and NIP film-coated electrodes were constructed to determine the apparent imprinting factor (IF). Then, the IF was calculated by dividing the slope of the calibration plot for the MIP film-coated electrode by the slope for the NIP film-coated electrode.

#### 2.4.3 Preparation of the Real Meat Sample

The pork sample preparation procedure is shown in Scheme 3. A sample of pork meat was purchased from a local market. The meat (5 g) was chopped and ground to a paste using a high-speed food blender. A 5% metaphosphoric acid and 20% acetonitrile solution sample (5 mL) were added to the meat paste and then stirred for 5 min. Next, acetonitrile (10 mL) was added to this mixture and stirred for another 10 min. The mixture was transferred to a centrifuge tube (100 mL) and centrifuged at 5000 rpm for 10 min. Afterward, the supernatant was collected. The residue was extracted the same way again, and the supernatants were combined. The collected supernatant was transferred to a glass dish and evaporated at 40 °C for 4 days. Subsequently, a methanol sample (5 mL) was used to dissolve the residue and then filtered on a Whatman chromatography paper. The filtered solution was collected in a flask, and its volume was decreased to 1 mL by evaporation at room temperature. Finally, the pork extract solution (1 mL) was spiked with a known amount, namely, 4.99 mg, of **7,8-DiMeIQx**, and then diluted with 10 mM KF to achieve the 1 mM concentration. This stock solution was afterward applied for testing the devised MIP-based chemosensor.

## 3 Results and Discussion

### 3.1 Designing Molecularly Imprinted Cavities in the MIP for Selective **7,8-DiMeIQx** Recognition

Choosing suitable functional monomers is crucial for molecular imprinting. Therefore, we aimed to generate molecular cavities in MIPs to mimic dsDNA in binding the HAA target analyte with interactions similar to those that occur during HAA intercalation in real dsDNA. For that, we molecularly modeled the structure of pre-polymerization complexes of the **7,8-DiMeIQx** template with selected functional monomers.

First, the molecular structures of the **Abt** and **Tbt** functional monomers and then the structures of their pre-polymerization complexes with the planar aromatic **7,8-DiMeIQx** template were thermodynamically optimized. Apparently, the template formed stable 1:1 complexes with both functional monomers. Moreover, the Gibbs free energy gain due to this complexation was most negative for the **Abt–Tbt**-(**7,8-DiMeIQx**) complex of the 2:1:1 stoichiometry (Scheme 1), indicating that the complex is most stable at this stoichiometry. Importantly, all generated possible pre-polymerization complexes were significantly more stable than the (Watson–Crick)-like **Abt–Tbt** complex (Table 1). However, the above comparison of complex stabilities should be taken with circumspection because corrections concerning the solvent nature and temperature should be considered. Significantly, former research revealed that intercalators bound to dsDNA with high affinity if *K*_s_ values were of the order of 10^4^ M^–1^.^36^ Evidently, the herein examined interactions of the **7,8-DiMeIQx** template with the **Abt** and **Tbt** monomers were strong, thus ensuring the formation of the stable pre-polymerization complex and successful **7,8-DiMeIQx** imprinting in the MIP.

**Table 1:** Gibbs Free Energy Change (Δ*G*) Accompanying the **Abt** and **Tbt** Functional Monomers Pairing and Forming Complexes with the **7,8-DiMeIQx** Template in Acetonitrile Calculated Using the DFT Method at the M06-2x/6-31G* Level with Dispersion Correction (D3)

complex	Δ*G*, kJ/mol
**Abt–Abt**	–17.2
**Tbt–Tbt**	–18.3
**(7,8-DiMeIQx)-(7,8-DiMeIQx)**	–22.3
**Abt**–**Tbt**	–26.4
**Tbt-(7,8-DiMeIQx)**	–34.4
**Abt-(7,8-DiMeIQx)**	–46.2
**Abt-(7,8-DiMeIQx)-Tbt**	–66.5
**Abt**–**Tbt/Abt-(7,8-DiMeIQx)**	–84.3

**Scheme 2:**
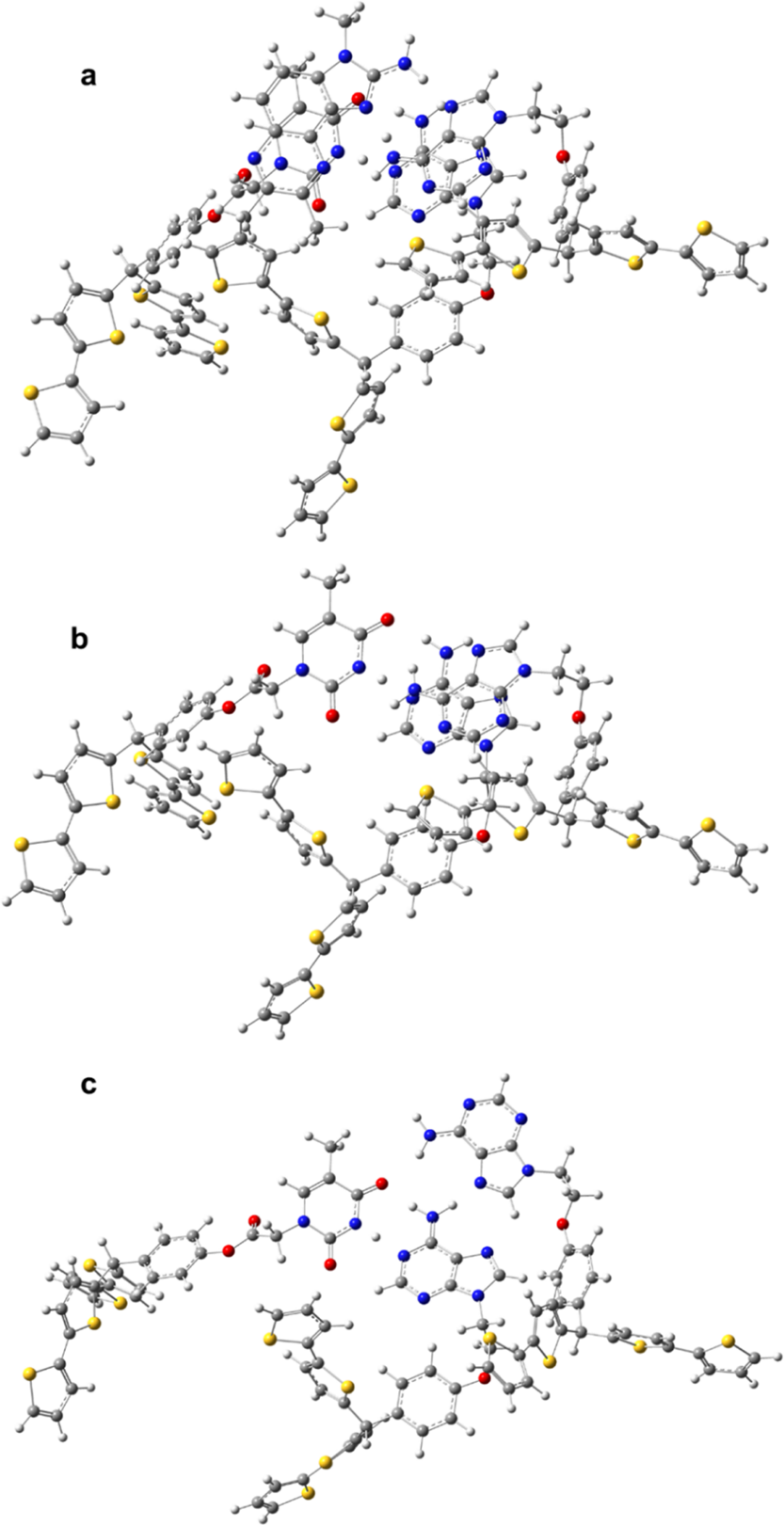
Structures Optimized by DFT at the M06-2X/6-31G* Level with Dispersion Correction (D3) for (a) the **Abt–Tbt/Abt**-(**7,8-DiMeIQx**) Pre-polymerization Complex and (b, c) the Molecular Cavity Imprinted in the MIP with the Template Molecule Removed in the (b) Ready for Analyte Recognition and (c) Inactive Conformations

Then, the pre-polymerization complex structure was optimized (Schemes 1a-c and 2a). This computational modeling indicated that the formation of the **Abt–Tbt/Abt**-(**7,8-DiMeIQx**) complex in solution was highly preferred (Table 1). Furthermore, six hydrogen bonds combined with π–π stacking resulted in a strongly negative gain of Gibbs free energy (Δ*G* = −84.3 kJ/mol) for this complexation.

Subsequently, the template molecule was removed from the structure-optimized **Abt–Tbt/Abt**-(**7,8-DiMeIQx**) complex, and then the terminal positions of bis(2,2′-bithien-5-yl)methane moieties were “frozen” to model interactions of the imprinted cavities with molecules of **7,8-DiMeIQx**. It occurred that these cavities might exist in two stable conformations (Schemes 1d,e and 2b,c) and that the Gibbs free energy of formation of the conformation capable of template binding was by 1.4 kJ/mol lower than that of the inactive conformation. With this behavior, the MIP film mimics allosteric molecular recognition in protein receptors.^31^

### 3.2 Synthesis of an MIP Film for **7,8-DiMeIQx** Determination

**Figure 1:**
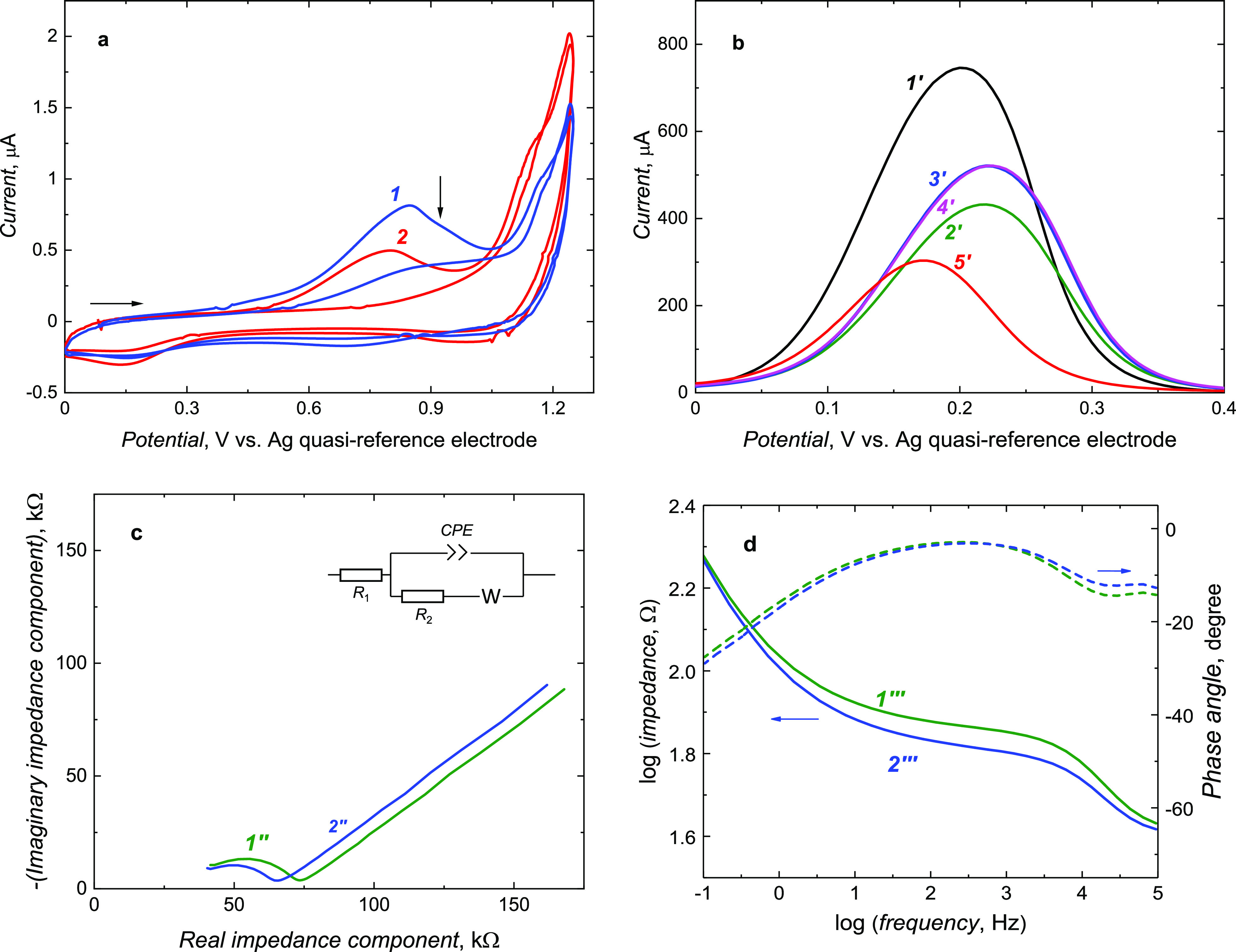
(a) Potentiodynamic curves for deposition by electropolymerization at 50 mV/s of the (*1*) MIP and (*2*) NIP film from the solution of 20 μM **7,8-DiMeIQx**, 40 μM **Abt**, 20 μM **Tbt**, 40 μM **Crl**, and 20 mM TBAClO_4_ in acetonitrile. (b) DPV curves for (*1′*) the bare and MIP film-coated on the 1 mm diameter Au disk electrode (*2′*) before and after **7,8-DiMeIQx** (*3′*) 20 min or (*4′*) 40 min extraction with 10 mM Et_3_N in acetonitrile and (*5′*) NIP film-coated. (c) Nyquist and (d) Bode plots of EIS spectra for the MIP film-coated electrode (*1″* and *1‴*) before and (*2″* and *2‴*) after **7,8-DiMeIQx** extraction. The DPV and EIS measurements were performed using 100 mM K_3_[Fe(CN)_6_] and 100 mM K_4_[Fe(CN)_6_] redox probes in PBS (pH = 7.4).

The **MIP**-[**Abt–Tbt**/**Abt**-(**7,8-DiMeIQx**)] film was synthesized from the pre-polymerization complex and simultaneously deposited on the electrodes by potentiodynamic electropolymerization. For that, **7,8-DiMeIQx**, **Abt**, **Tbt**, and **Crl** served as the template, two functional monomers, and the cross-linking monomer, respectively. The MIP film was deposited by performing two potentiodynamic cycles between 0 and 1.25 V versus an Ag quasi-reference electrode at 50 mV s^–1^ (Figure 1a). In the first cycle, a well-developed anodic peak current appeared at 0.85 V versus an Ag quasi-reference electrode. This peak was significantly lower in the second scan. In the NIP deposition, this peak was by 50 mV shifted to lower potentials. This peak potential difference may be attributed to stable **Abt–Tbt**/**Abt**-(**7,8-DiMeIQx**) complex formation.^37^ Notably, for NIPs, the Faradaic current of the monomers in the second scan decreased much more significantly, indicating the formation of a more compact film. The **7,8-DiMeIQx** template was removed from the MIP film by 20 min extraction with 10 mM Et_3_N in acetonitrile.

The successful MIP film deposition on the electrode surface was confirmed with DPV by recording curves for the solution of 100 mM K_3_[Fe(CN)_6_] and 100 mM K_4_[Fe(CN)_6_] redox probes in PBS, pH = 7.4 (Figure 1b). After the electropolymerization, the redox probe anodic peak decreased significantly compared to that at the bare electrode, proving successful electrode coating. Moreover, after 20 min extraction, the DPV redox probe peak increased. If extraction was performed for a longer time, this peak remained constant (data not shown), suggesting that the extraction was completed after 20 min. Moreover, both the MIP and NIP film-coated electrodes were characterized by EIS. Flattened semicircles corresponding to the charge transfer resistance are present in both Nyquist plots (Figure 1c). This semicircle diameter dropped slightly after template extraction. Moreover, the straight line in the low-frequency part of the plot originating from Warburg diffusion was not affected by MIP film deposition nor template extraction. In the Bode plot (Figure 1d), a pronounced change in impedance and a slight decrease in phase angle in the 1 to 10 000 Hz and 10 to 100 kHz frequency range, respectively, were seen.

### 3.3 Spectroscopic and Microscopic Characterization of the MIP and NIP Films

The deposited MIP and NIP films were characterized by FTIR spectroscopy (Figure S1 in the Supporting Information) and XPS (Table S1 in the Supporting Information). Both techniques confirmed successful film deposition. The FTIR spectrum of the MIP film after template extraction (curve 2 in Figure S1) is similar to that for the NIP film (curve 3 in Figure S1). However, a 1600–1700 cm^–1^ band is more pronounced for the MIP film before extraction (curve 3 in Figure S1). This band may be attributed to the C=C and C=N bond stretching of the **7,8-DiMeIQx** template present in the MIP film after electropolymerization.

Because of no characteristic elements in the **7,8-DiMeIQx** template, it was impossible to confirm the presence of the template before and then its absence after extraction from the MIP by the XPS analysis. However, a slight increase in the oxygen-to-nitrogen atomic content ratio after template extraction may suggest the extraction because oxygen is absent in **7,8-DiMeIQx** (Table S1). Furthermore, the oxygen-to-nitrogen atomic ratio in the MIP film before extraction is lower than that for both MIP after extraction and NIP films.

Moreover, the MIP and NIP films were AFM imaged (Figure S2). Spherical grains fused into rounded flattened “pancake-like” structures of 40 to 120 nm in diameter are seen in both films. The size of these structures did not change after template extraction. However, then, the MIP and NIP film’s roughness slightly increased and decreased, respectively. Both films were very thin (Table S2). Therefore, their thickness was not affected by template extraction.

### 3.4 **7,8-DiMeIQx** Determination with the MIP and NIP Film-Coated Electrodes

The template-extracted MIP film-coated electrodes were applied for **7,8-DiMeIQx** determination. The MIP film-coated electrode characterization (Figure 1b) suggested that the DPV peak current in the presence of the ferrocene redox probe in the solution should depend on the **7,8-DiMeIQx** concentration because of the so-called “gate effect”.^38,39^ That is, it is assumed that the MIP cavities binding of the analyte molecules causes film swelling or shrinking, thus affecting redox probe diffusion to the electrode surface. Moreover, a recently reported study suggests that the “gate effect” mechanism is different for conductive MIP film-coated electrodes.^40^ That is, a drop in the Faradaic current of the redox probe originates from the drop in polymer film conductivity because of analyte binding and not because of hindering the diffusion of the redox probe. Indeed, the DPV peak current decreased with the **7,8-DiMeIQx** concentration increase (Figure S3), and this decrease was linear (Figure S4).

The main reason for the MIP film deposition is to incur high selectivity of the chemosensor devised. However, the present DPV chemosensor selectivity, determined from the slope of the calibration curve for **7,8-DiMeIQx** to that for a common biological interferent, namely, glucose, urea, and creatinine, was insufficient despite a high apparent imprinting factor of IF = 23.

**Figure 2:**
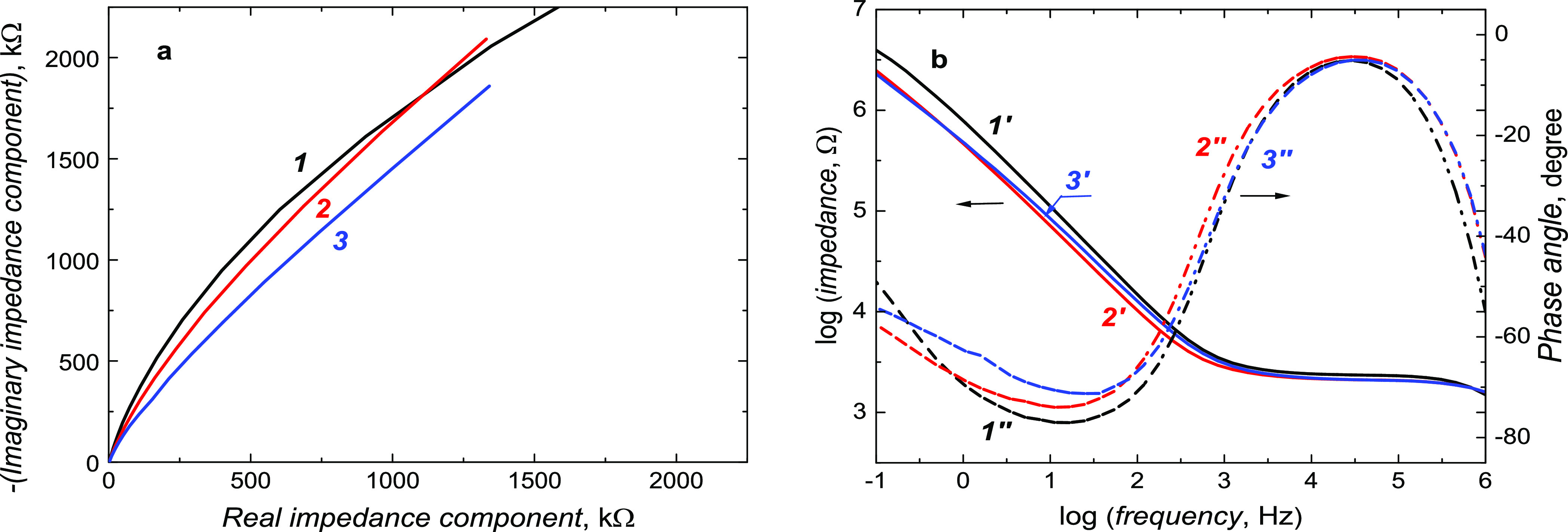
(a) Nyquist and (b) Bode plots of the EIS spectra recorded for the MIP film-coated electrodes (*1*, *1′*, and *1″*) before and (*2*, *2′*, and *2″*) after **7,8-DiMeIQx** template extraction and (*3*, *3′*, and *3″*) in 105.3 μM **7,8-DiMeIQx**. The measurements were performed using a 1 mm diameter Au disk electrode in 10 mM KF.

**Figure 3:**
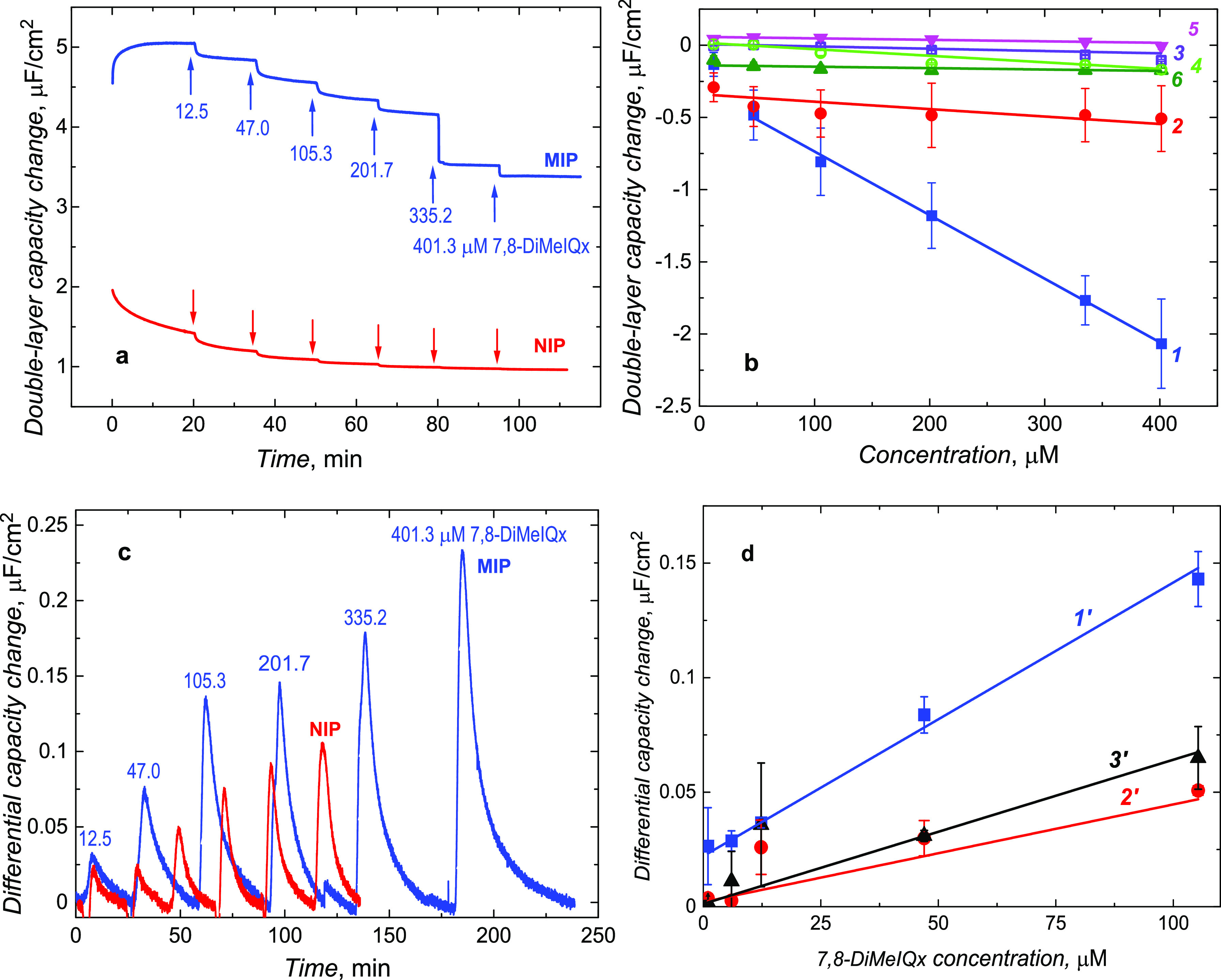
Double-layer capacity changes with time for the MIP and NIP film-coated electrodes under (a) steady-state solution and (c) FIA conditions. (b) Calibration plots of double-layer capacity changes under steady-state solution conditions at the (*1*, *3*–*6*) MIP or (2) NIP film-coated electrodes. The calibration plots were constructed for different concentrations of (*1*, *2*) **7,8-DiMeIQx**, (*3*) glucose, (*4*) urea, (*5*) creatinine, and (*6*) **8-MeIQx—**a close structural analogue of the target analyte. (d) Calibration plots of double-layer capacity changes under FIA conditions at the (*1′*) MIP and (*2′*) NIP film-coated and (*3′*) bare Pt electrodes. The calibration plots were constructed for different concentrations of **7,8-DiMeIQx**. The measurements were performed with a 1 mm diameter (a, b) Au or (c, d) Pt disk electrode using 10 mM KF, at a 0.21 V vs an Ag quasi-reference electrode, 10 mV amplitude, and 500 Hz frequency of potential changes. All data points for calibration plots were recorded thrice to calculate each presented point’s average value and standard deviation.

This unfavorable performance necessitated the use of another transduction technique, which would afford sufficient selectivity of determination. Toward that, the EIS spectra were recorded at the template-extracted MIP film-coated electrode in 10 mM KF at 0.21 V versus an Ag quasi-reference electrode. At this low potential, no electrode process occurred (Figure 2a). Moreover, in the Bode plot (Figure 2b), the phase angle in the 100 to 1000 Hz frequency range significantly changed after **7,8-DiMeIQx** extraction, and it returned to its original value after **7,8-DiMeIQx** binding. Therefore, the double-layer capacity change (Δ*C*_dl_) with the analyte or interference concentration change in 10 mM KF was measured using capacitive impedimetry (CI) under both FIA and steady-state solution conditions at 500 Hz frequency of potential changes and 10 mV potential amplitude (Figure 3a-3d).

Under steady-state solution conditions, the double-layer capacity change linearly decreased with the **7,8-DiMeIQx** concentration increase from 47 to 400 μM (Figure 3a,b) for both MIP and NIP films. Importantly, for both the MIP and NIP film-coated electrodes, the response was in the direction opposite to that for the bare electrode (Figure S5). This effect resulted in a pronounced apparent imprinting factor and selectivity of the CI chemosensor. Moreover, a significantly broader chemosensor linear dynamic concentration range under steady-state conditions, that is, when the time is sufficiently long to reach partitioning equilibrium, may suggest that the number of imprinted cavities engaged in **7,8-DiMeIQx** entrapping is much higher than that under FIA conditions. The MIP chemosensor response under the steady-state conditions follows the linear regression equation Δ*C*_dl_ [μF cm^–2^ μM^–1^] = −2.96 × 10^–1^ (±2.28 × 10^–2^)–4.4 × 10^–3^ (±9.56 × 10^–5^) *c*_**7,8-DiMeIQx**_ [μM] with the regression coefficient *R*^2^ = 0.9986. The LOD determined at *S*/*N* = 3 and apparent imprinting factor was LOD = 15.5 μM and IF = 8.5, respectively. The CI chemosensor response to glucose, urea, and creatinine interferences was less pronounced than to **7,8-DiMeIQx**. The calculated selectivity was 27.5, 9.7, and 43, respectively. It appeared that the chemosensor response to an **8-MeIQx** close structural analogue of the **7,8-DiMeIQx** analyte was negligible.

Moreover, the **7,8-DiMeIQx** determinations were performed under FIA conditions using a specially designed flow cell.^35^ For that, first, the **MIP** and **NIP** films were deposited, under steady-state conditions, on the Pt electrodes in a three-electrode cell. Then, after template extraction, the electrodes were mounted in the flow cell and used for the **7,8-DiMeiQx** determination under optimized conditions of the 50 μL/min flow rate of the 10 mM KF carrier solution. Surprisingly, the double-layer capacity of both films increased under these conditions with the **7,8-DiMeiQx** concentration increase (Figure 3c). However, the peaks were higher at the MIP film-coated electrode, and the signal returned to its baseline slowly, suggesting that the **7,8-DiMeiQx** affinity to the MIP was higher than to the control NIP. Furthermore, for both films, the shape of the peaks was non-gaussian, indicating that the time was too short for reaching the partitioning equilibrium. Moreover, the peaks were significantly smaller at a lower flow rate (Figure S6). Presumably, two processes occur on the MIP film-coated electrode. One, which was faster, involving the **7,8-DiMeIQx** adsorption on the film surface, caused an increase in the double-layer capacity. The other, which was slower, related to polymer shrinking or expelling ions from the film due to the **7,8-DiMeIQx** binding to molecularly imprinted cavities located deeper inside the film, caused the opposite effect, that is, the capacity decrease. This latter effect would then be dominant in the steady-state experiments.

Under FIA conditions, the linear dynamic concentration range of the MIP chemosensor extends from 1 to 100 μM **7,8-DiMeIQx**. Moreover, the CI chemosensor response obeys the linear regression equation in the form of Δ*C*_dl_ [μF cm^–2^ μM^–1^] = 2.21 × 10^–2^ (±1.23 × 10^–3^) + 1.19 × 10^–3^ (±5.44 × 10^–5^) *c*_**7,8-DiMeIQx**_ [μM] with the regression coefficient and LOD at *S*/*N* = 3 of *R*^2^ = 0.994 and LOD = 3.1 μM, respectively. The apparent imprinting factor was determined as IF = ∼2.8. This value is much lower than that determined under steady-state conditions, thus supporting the inference that only the outer part of the MIP film participated in the analyte binding in the FIA analysis. Interestingly, the NIP film-coated Pt electrode response to **7,8-DiMeIQx** was nearly indistinguishable from that of the bare Pt electrode (Figures 3d and S7). Therefore, the exact value of the apparent imprinting factor cannot reasonably be determined. However, it appeared that analyte binding in NIP is relatively small. Moreover, for **7,8-DiMeIQx** concentrations exceeding 100 μM, the MIP imprinted cavities seem to be saturated (Figure S7). Therefore, Langmuir–Freundlich (LF) isotherms were fitted to the sorption data acquired and the isotherm parameters calculated (Table S3). The LF isotherm fitted quite well the data points recorded for the MIP film-coated electrode. Importantly, homogeneity factor, *n*, calculated for the MIP film-coated electrode was significantly lower than those recorded for the bare and NIP film-coated electrodes equaling 0.67. Evidently, both processes were visible: the selective **7,8-DiMeIQx** binding in MIP cavities and non-specific binding on the MIP film surface.

### 3.5 **7,8-DiMeIQx** Determination in Extracts of Pork Meat Samples

**Scheme 3:**
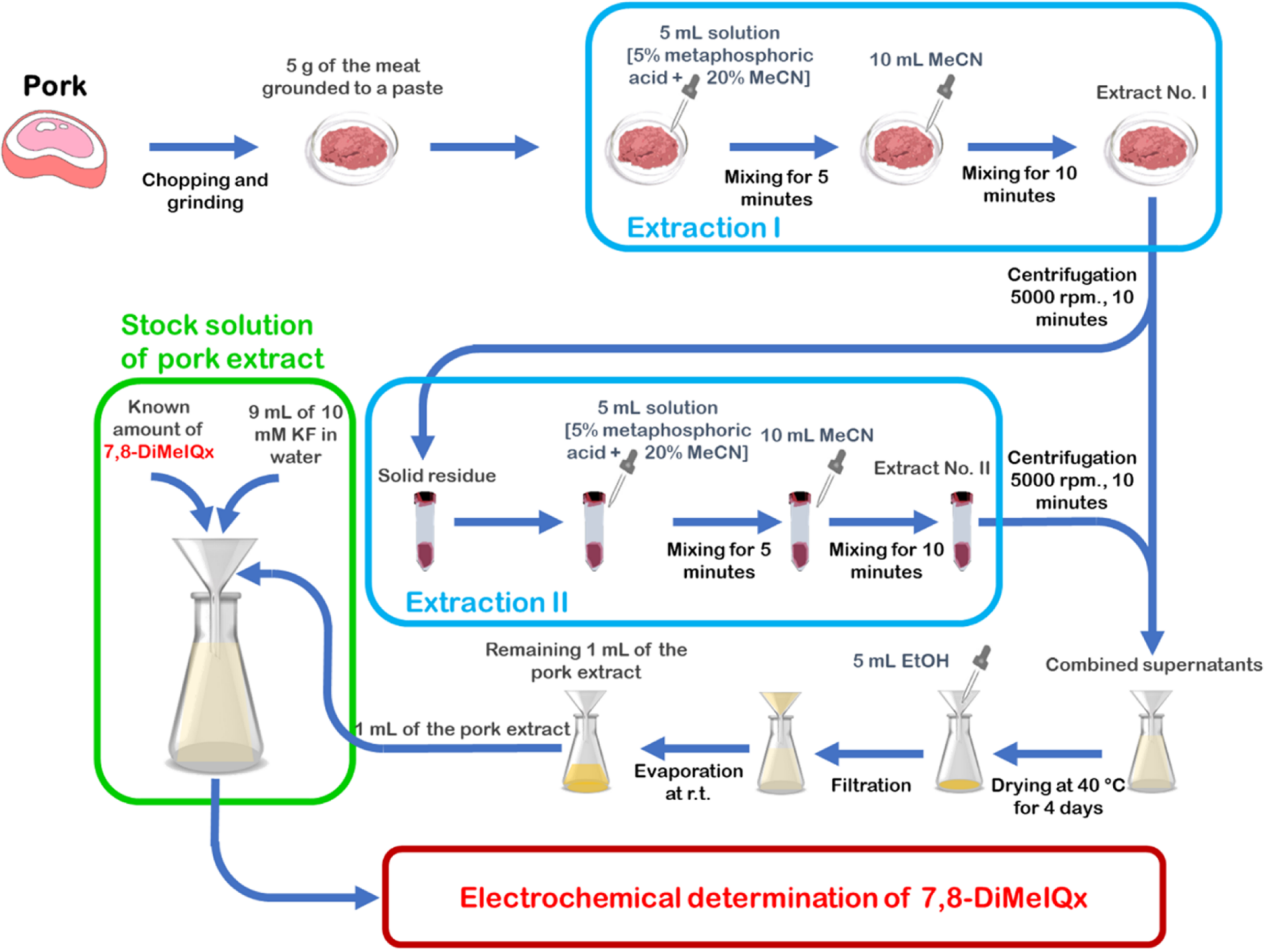
Procedure of the Pork Meat Sample Preparation for the Determination of **7,8-DiMeIQx** Using an MIP Film-Coated Electrode

The applicability of the MIP chemosensor for HAAs determination in real samples was demonstrated with the example of **7,8-DiMeIQx** sensing in spiked pork meat extract samples (Scheme 3). The samples were prepared according to a slightly modified procedure that was reported elsewhere.^41^ The matrix effect was visible, and the recorded responses were slightly higher than those for the blank KF solution (Figure S9). However, the recovery was quite appreciable (Table 2), confirming that the MIP film-coated electrode is suitable for the **7,8-DiMeIQx** determination in real food samples.

**Table 2:** **7,8-DiMeIQx** Determination in Pork Meat Extract

sample no.	added **7,8-DiMeIQx** concentration, μM	measured double-layer capacity change, nF cm^–2^	determined **7,8-DiMeIQx** concentration, μM	recovery, %
1	47.0	–0.52 (±0.27)	51.3 (±4.9)	109.1 (±10.4)
2	105.3	–0.84 (±0.28)	124.1 (±2.8)	117.8 (±2.6)
3	201.7	–1.16 (±0.18)	195.2 (±27)	96.8 (±13.4)
4	335.2	–1.51 (±0.34)	276.9 (±10.1)	82.6 (±3.0)
5	401.3	–1.93 (±0.35)	370.7 (±12.9)	92.4 (±3.2)

In summary, an MIP chemosensor selective to **7,8-DiMeIQx** was successfully devised, fabricated, and tested. The **7,8-DiMeIQx** analyte was determined using CI under both FIA and steady-state solution conditions. Interestingly, MIP film-coated electrode capacity changes under FIA were opposite to those under steady-state solution conditions. Presumably, two processes governed these changes. The fast one, ascribed to **7,8-DiMeIQx** adsorption, caused an increase in the double-layer capacity. The other, slow, most likely related to polymer shrinking or expelling ions from the film because of the **7,8-DiMeIQx** binding to molecularly imprinted cavities located deeper in the polymer film, caused the opposite effect, that is, the capacity decrease. Moreover, steady-state solution conditions occurred to be superior to those of FIA. That is, the MIP film-coated electrode responded to **7,8-DiMeIQx** concentration changes in a much broader linear dynamic concentration range (47–400 μM) than for the former. The estimated apparent imprinting factor was very high, IF = 8.5. Therefore, the MIP film-coated electrode was successfully applied for **7,8-DiMeIQx** determination in pork meat under steady-state solution conditions.

## Supporting Information Available

IR and XPS characterization as well as AFM imaging of the MIP and NIP films, EIS characterization of the MIP and NIP films, DPV determination of **7,8-DiMeIQx**, additional impedimetric measurements, and isotherm fitting (PDF)
