# Supplementary material for: Selective Impedimetric Chemosensing of Carcinogenic Heterocyclic Aromatic Amine in Pork by dsDNA-Mimicking Molecularly Imprinted Polymer Film-Coated Electrodes
Source: J Agric Food Chem. 2021 Nov 29;69(48):14689–98. doi: 10.1021/acs.jafc.1c05084 (PMC8662733; doi:10.1021/acs.jafc.1c05084)
Supplement: Supplementary file 1 — jf1c05084_si_001.pdf [file jf1c05084_si_001.pdf]

Supporting Information
for
Selective impedimetric chemosensing of carcinogenic heterocyclic aromatic amine by dsDNA mimicking molecularly imprinted polymer film-coated electrodes

Viknasvarri Ayerdurai,[a] Alvaro Garcia-Cruz,[a,†] Joanna Piechowska,[a] Maciej Cieplak,[a,*] Paweł Borowicz,[a] Krzysztof R. Noworyta,[a] Grzegorz Spolnik,[b] Witold Danikiewicz,[b] Wojciech Lisowski,[a] Agnieszka Pietrzyk-Le,[a] Francis D'Souza,[c,*] Wlodzinierz Kutner[a,d,*] and Piyush Sindhu Sharma[a]

[a] *Institute of Physical Chemistry, Polish Academy of Sciences, Kasprzaka 44/52, 01-224 Warsaw, Poland.*

[b] *Institute of Organic Chemistry, Polish Academy of Sciences, Kasprzaka 44/52, 01-224 Warsaw, Poland.*

[c] *Department of Chemistry, University of North Texas, 1155 Union Circle No. 305070, Denton, TX 76203-5017, United States*

[d] *Faculty of Mathematics and Natural Sciences. School of Sciences, Cardinal Stefan Wyszynski University in Warsaw, Wóycickiego 1/3, 01-938 Warsaw, Poland.*

[†] Present address: *Biotechnology Group, Department of Chemistry, University of Leicester, Leicester, United Kingdom.*

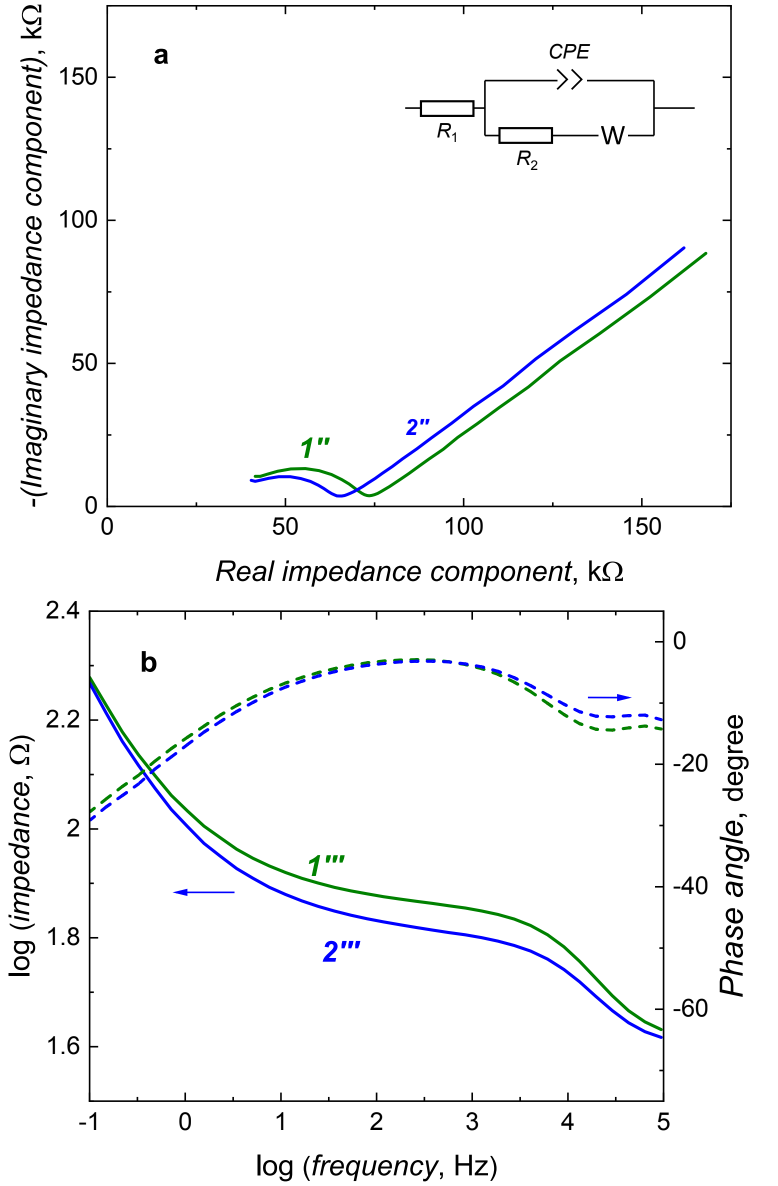

Figure S1. (a) Nyquist and (b) Bode plots of EIS spectra for MIP film-coated electrode (*1″* and *1‴*) before and (*2″* and *2‴*) after **7,8-DiMeIQx** extraction. The EIS measurements were performed using 100 mM $K_3[Fe(CN)_6]$ and 100 mM $K_4[Fe(CN)_6]$ redox probe PBS (pH = 7.4).

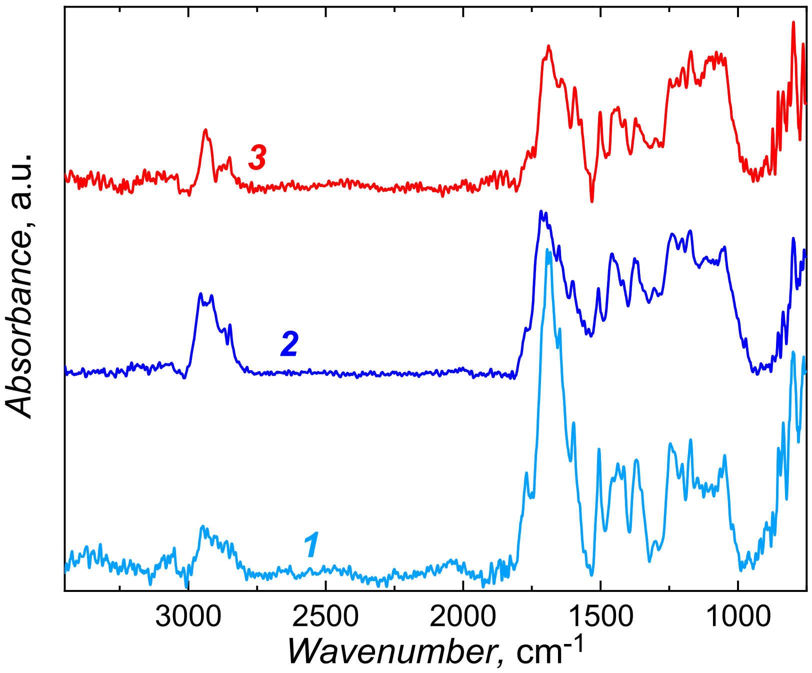

Figure S2. (a) FTIR spectra for Au-layered glass slides coated with the MIP film (*1*) before and (*2*) after template extraction with 10 mM Et_3N in acetonitrile, and (*3*) NIP film.

Table S1. XPS determined the atomic content of the MIP and NIP film.

Element	Atomic content, %			
	**MIP before template extraction**	**MIP after template extraction**	**NIP before template "extraction"**	**NIP after template "extraction"**
**O**	6.09	7.77	6.44	7.36
**N**	3.95	4.50	3.60	3.65
**C**	80.04	78.31	78.47	78.43
**S**	9.92	9.42	11.49	10.56

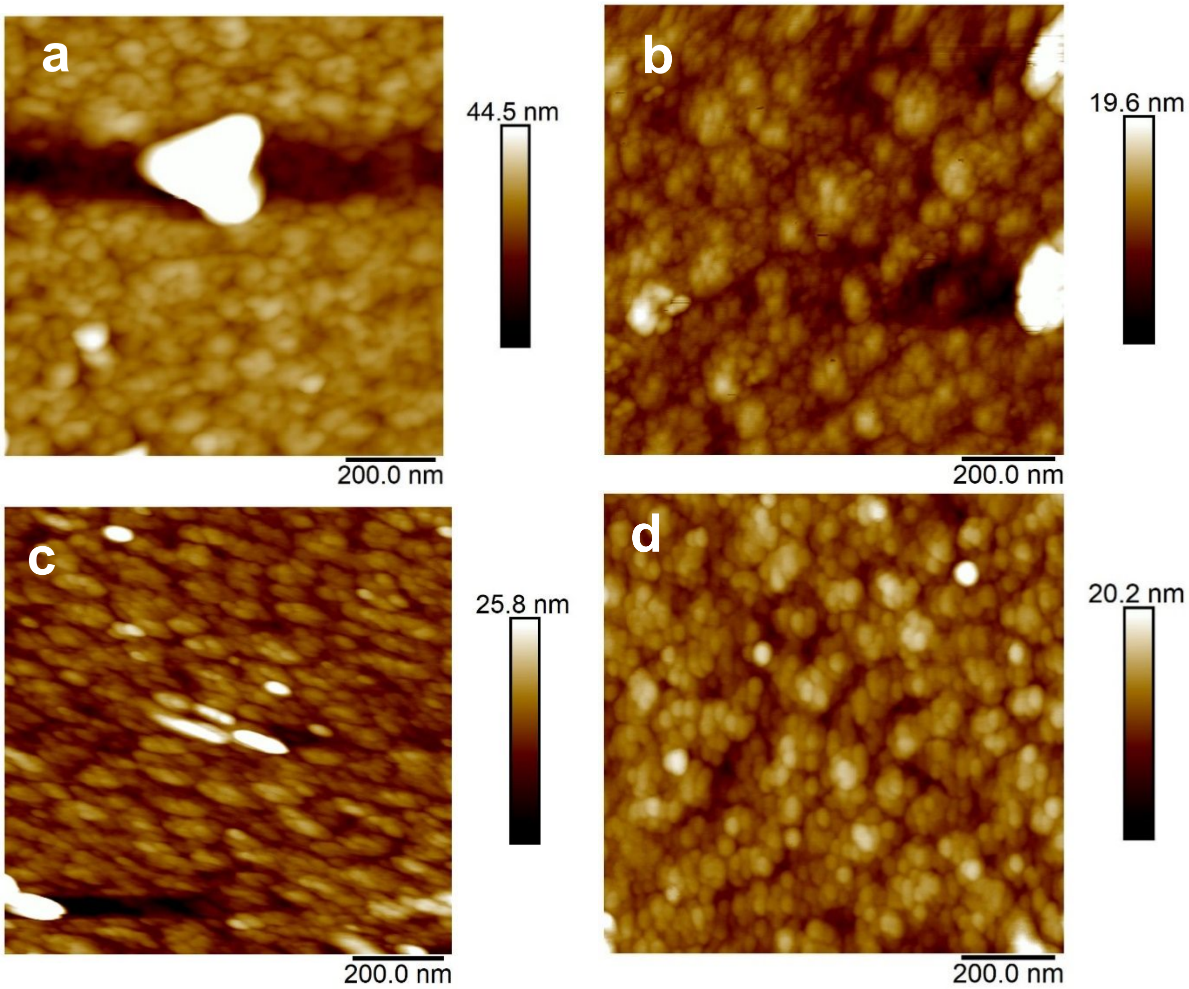

Figure S3. AFM images of the (a and b) MIP and (c and d) NIP films, (a and c) before and (b and d) after **7,8-DiMeIQx** template extraction.

Table S2. AFM imaging determined MIP and NIP film properties.

**Film property**	**MIP before template extraction**	**MIP after template extraction**	**NIP before film rinsing**	**NIP after film rinsing**
Thickness, nm	9.4 ± 3.0	7.5 ± 1.2	8.4 ± 1.9	12.0 ± 2.6
Roughness, R_a, nm	2.2 ± 0.9	4.2 ± 1.5	6.4 ± 1.4	3.7 ± 0.9
Grain diameter, nm	85.0 ± 35.0	85.0 ± 43.0	82.0 ± 35.0	88.0 ± 36.0
Phase, degree	111.0 ± 1.0	23.3 ± 0.4	19.4 ± 0.9	4.9 ± 0.1

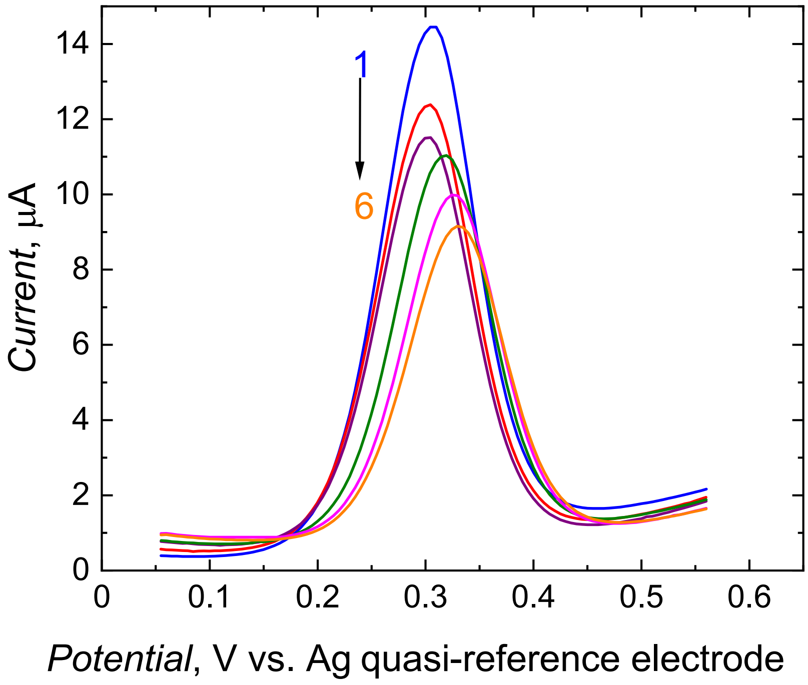

Figure S4. DPV curves recorded at the MIP film-coated 1-0 mm diameter Au disk electrode for the acetonitrile solution of 1 mM ferrocene, 0.1 M $(TBA)ClO_4$, and (*1*) 0, (*2*) 2.5 , (*3*) 5.0, (*4*) 7.4, (*5*) 9.8, and (*6*) 12 µM **7,8-DiMeIQx**.

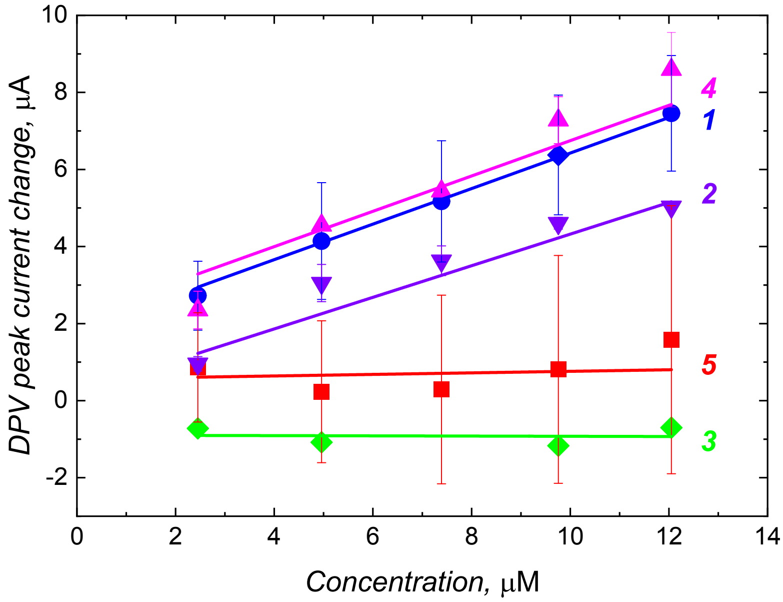

Figure S5. The DPV peak current calibration plots for acetonitrile solutions of 1 mM ferrocene, 0.1 M $(TBA)ClO_4$, and (*1*) **7,8-DiMeIQx**, (*2*) glucose, (*3*) urea, and (*4*) tyramine at the MIP film-coated and (*5*) **7,8-DiMeIQx** at the NIP film-coated 1-mm diameter Au disk electrode.

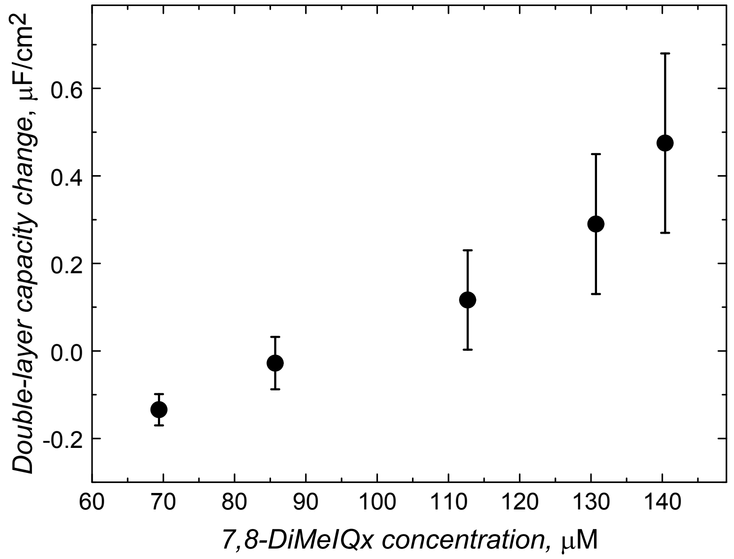

Figure S6. The calibration plot of the double-layer capacity change with the **7,8-DiMeIQx** concentration change at a bare 1-mm diameter Au disk electrode in 10 mM KF at 0.21 V vs. Ag quasi-reference with the 10-mV amplitude and 500-Hz ac voltage.

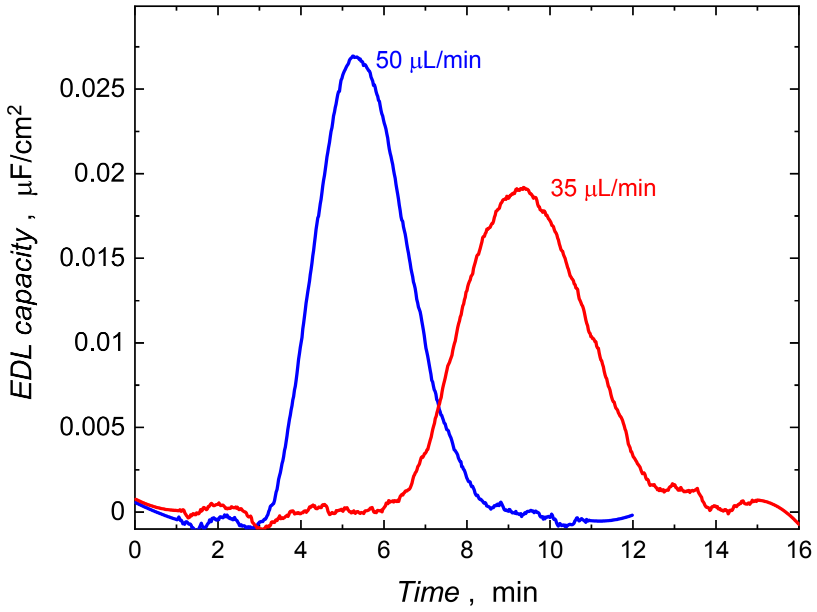

Figure S7. Double-layer capacity changes with time for the MIP film-coated electrode, recorded under FIA conditions for two different flow rates indicated at curves. The measurements were performed using a 1-mm diameter Pt disk electrode in 10 mM KF at 0.21 V vs. Ag quasi-reference with a 10-mV amplitude and 500 Hz frequency of potential changes.

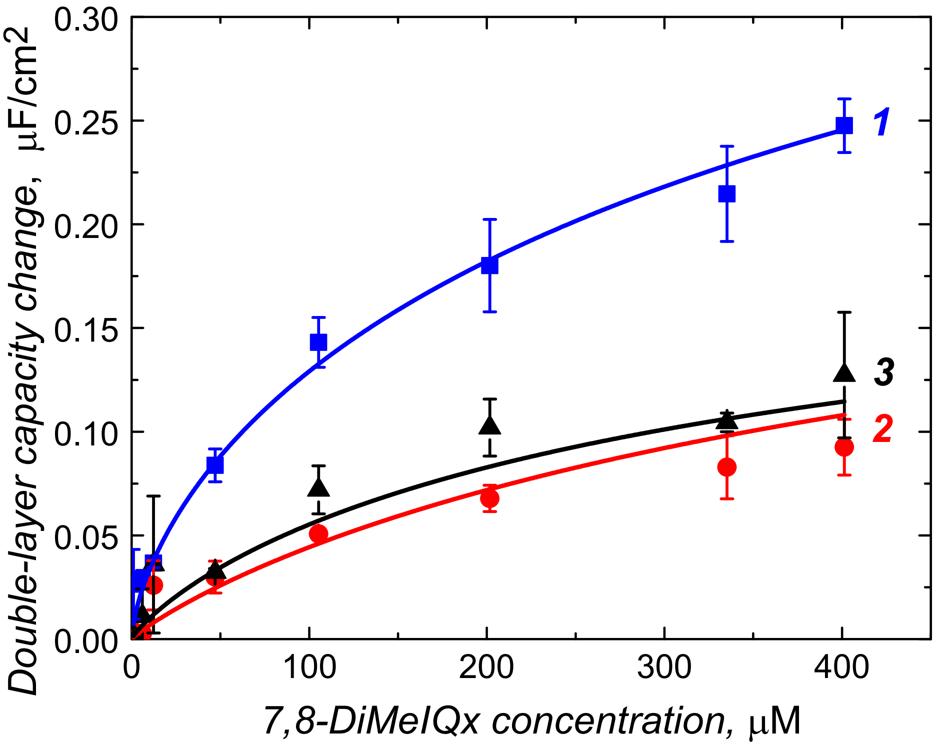

Figure S8. The double-layer capacity change dependence on the **7,8-DiMeIQx** concentration for the (*1*) MIP, (*2*) NIP film-coated, or (*3*) bare 1-mm diameter Pt disk electrode in 10 mM KF at 0.21 V vs. Ag/AgCl with the 10-mV amplitude, 500-Hz ac voltage, and a 50-µL/min flow rate.

Table S3. Calculated parameters of the Langmuir-Freundlich isotherms[a].

**Pt disk electrode**	**$\Delta C_{DL,max}$, µF/cm²**	**K, µM**	***n***	**R^2**
MIP film-coated	0.59 (±0.29)	1.49×10^{-3} (±1.86×10^{-3})	0.67 (±0.08)	0.991
NIP film-coated	0.26 (±0.68)	1.73×10^{-3} (±7.32×10^{-3})	0.90 (±0.29)	0.774
Bare	0.22 (±0.093)	2.82×10^{-3} (±2.74×10^{-3})	0.85 (±0.15)	0.994

[a] The Langmuir-Freundlich isotherm equation: $\Delta C_{\mathrm{DL}} = \Delta C_{\mathrm{DL,\,max}}\frac{(Kc)^n}{1+(Kc)^n}$
where $\Delta C_{DL,max}$, K, c, and n is the maximum double-layer capacity change, Langmuir-Freundlich constant, **7,8-DiMeIQx** concentration, and homogeneity factor, respectively.

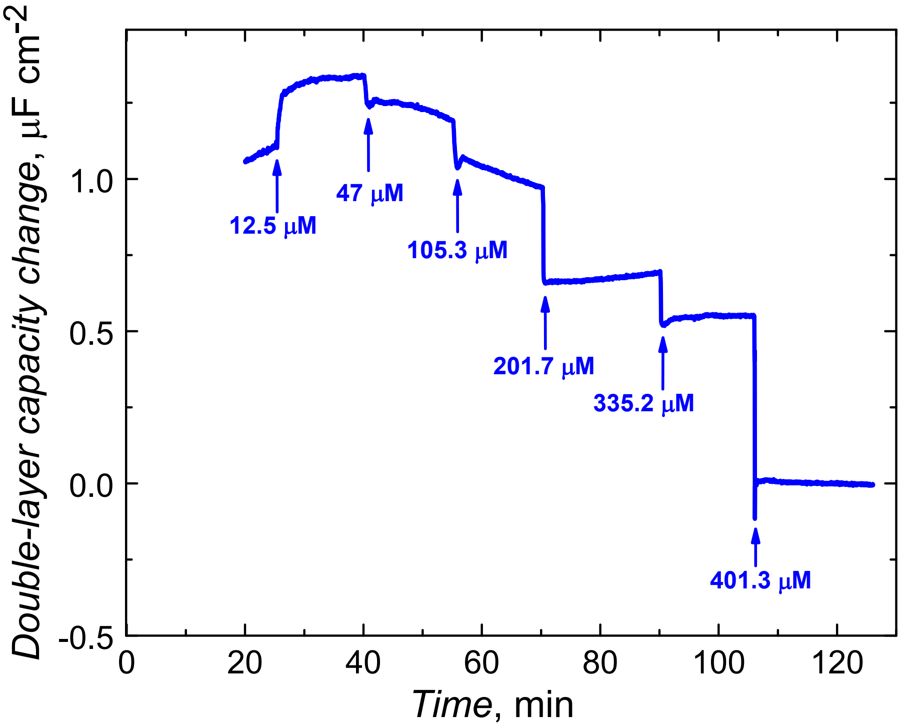

Figure S9. The double-layer capacity change with time for the MIP film-coated electrode under FIA conditions. The measurement was performed with a 1-mm diameter Au disk electrode using 10 mM KF, at 0.21 V vs. Ag quasi-reference electrode, 10-mV amplitude, and 500-Hz frequency.
